# Patent foramen ovale, bone cement implantation syndrome and paradoxical embolism: Case study and pathophysiological aspects

**DOI:** 10.1007/s00101-026-01706-x

**Published:** 2026-06-19

**Authors:** Ludwig Brandt, Ulrike Artmeier-Brandt, Klara Luisa Brandt

**Affiliations:** 1 abcGbR, Ernst-Udet-Straße 9, 85764 Oberschleißheim, Germany; 2 University Witten/Herdecke, Witten, Germany; 3 Faculty of Medicine, Vilnius University, Vilnius, Lithuania

**Keywords:** Middle cerebral artery, Atrial shunt, Perioperative ischemic stroke, Pulmonary vascular resistance (PVR), Total hip arthroplasty (THA), Arteria cerebri media, Atrialer Shunt, Perioperativer ischämischer Schlaganfall, Pulmonalvaskulärer Widerstand, Totaler Hüftgelenkersatz

## Abstract

Patients with a patent foramen ovale (PFO) have an increased risk of perioperative cerebral stroke due to the potential for paradoxical embolism. It is pathophysiologically plausible that this risk is further increased when bone cement implantation syndrome (BCIS) occurs during endoprosthetic joint surgery; however, despite double-digit prevalence rates for both PFO and BCIS, the perioperative concurrence of these conditions with subsequent paradoxical embolism has not previously been reported.

We describe an 81-year-old woman with a known PFO who developed intraoperative BCIS during cemented hip arthroplasty and subsequently sustained a paradoxical embolic stroke involving a large cerebral vessel. The potential pathophysiological links between PFO and BCIS are discussed, and anesthesiological implications and practical recommendations are derived from the current literature.

Published estimates of paradoxical embolism risk during endoprosthetic joint replacement are highly inconsistent, ranging from no increased risk to a 29-fold increase. This variability may partly reflect that many embolic events remain clinically silent.

For paradoxical embolism two conditions are required: (1) gaseous or particulate emboli must enter the venous circulation and (2) the physiological left-to-right interatrial shunt must reverse to a right-to-left shunt through the PFO. Both mechanisms can be promoted by BCIS by generating embolic load from the surgical field and increasing pulmonary vascular resistance, thereby favoring right-to-left shunting. Because specific anesthesiological preventive and therapeutic options are limited, close postoperative monitoring is essential and any new neurological or hemodynamic abnormalities should prompt immediate comprehensive diagnostic evaluation.

**Graphic abstract:**

**Figure:**
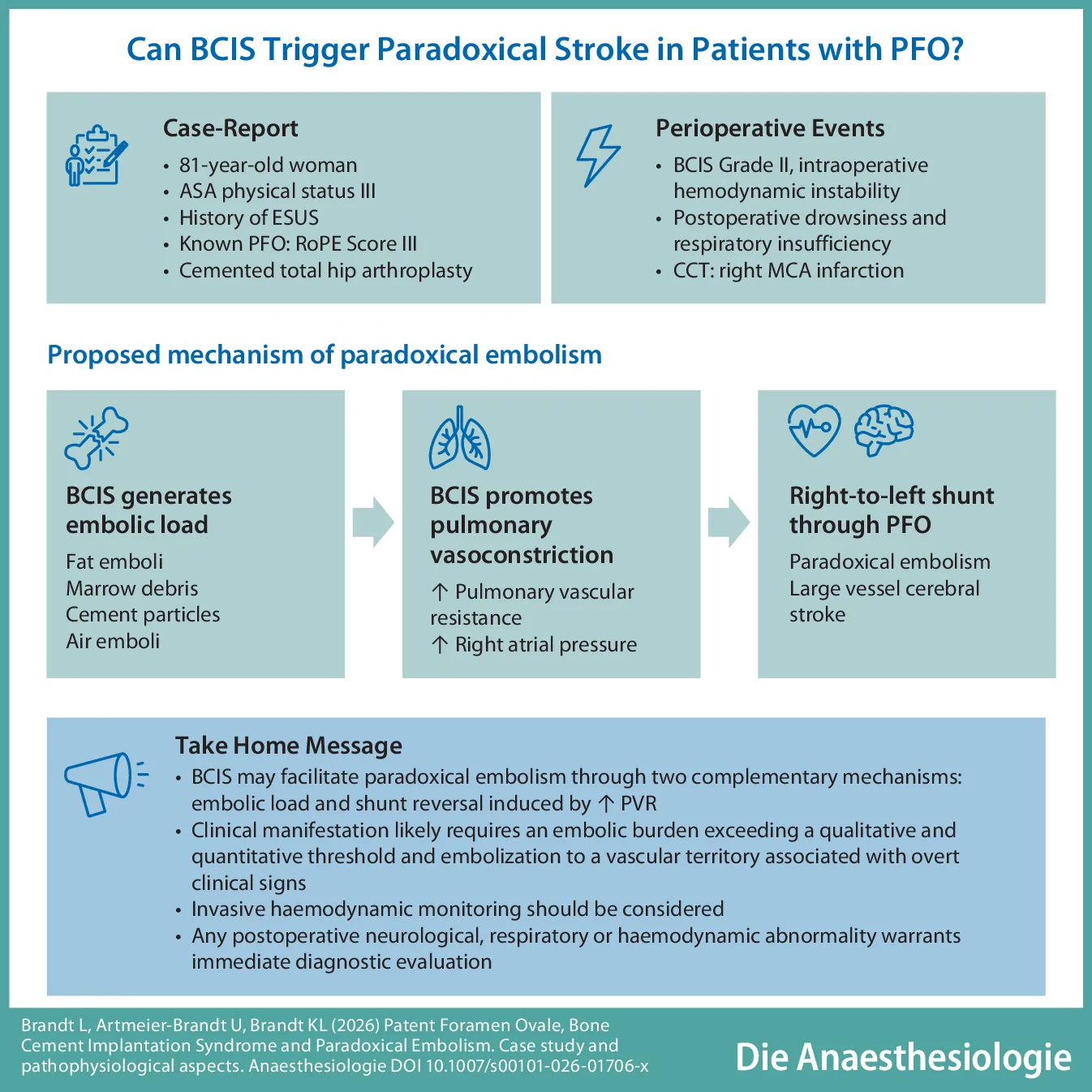

## Abstract (de)

Patienten mit einem offenen Foramen ovale (PFO) haben wegen der Gefahr einer paradoxen Embolie ein erhöhtes Risiko für einen perioperativen cerebralen Insult. Es erscheint pathophysiologisch plausibel, dass dieses Risiko durch Auftreten einer Knochenzementreaktion („bone cement implantation syndrome“, BCIS) bei endoprothetischen Gelenkoperationen weiter zunimmt. Aber trotz einer PFO- bzw. BCIS-Inzidenz im jeweils zweistelligen Prozentbereich gibt es bisher keine Berichte über die mögliche Koinzidenz beider Komplikationen.

Am Beispiel einer 81-jährigen PFO-Trägerin, die während einer Hüfttotalendoprothese (Hüft-TEP) ein BCIS erlitt und sich in der Folge eine paradoxe Embolie mit zerebralem Insult entwickelte, werden die pathophysiologischen Interaktionen beider Krankheitsbilder aufgezeigt. Anhand der aktuellen Literatur werden die daraus folgenden anästhesiologischen Implikationen diskutiert und Empfehlungen abgeleitet.

Die Wahrscheinlichkeit einer paradoxen Embolie bei endoprothetischem Gelenkersatz wird im Schrifttum mit großer Inkohärenz angegeben, von 0 % bis zu einem um den Faktor 29 gesteigerten Risiko. Diese schwer vereinbarenden Befunde lassen sich dadurch erklären, dass nicht alle stattfindenden Embolien auch klinisch sichtbar werden.

Für eine paradoxe Embolie sind zwei Voraussetzungen notwendig: Im venösen Gefäßsystem müssen gasförmige oder korpuskuläre Emboli vorhanden sein, und es muss es zu einer Umkehr des physiologischen transforaminalen Links(L)/Rechts(R)-Shunts zu einem pathologischen R/L-Shunt kommen. Beide Mechanismen werden durch ein BCIS induziert, im Operationsgebiet werden Emboli freigesetzt und durch einen Anstieg des pulmonalvaskulären Widerstands (PVR) entsteht ein R/L-Shunt. Die Gefahr einer paradoxen Embolie nimmt zu. Die anästhesiologischen prophylaktischen und therapeutischen Möglichkeiten zur Intervention sind begrenzt. Bei postoperativen neurologischen oder hämodynamischen Auffälligkeiten ist deshalb eine sofortige umfassende Diagnostik zur Abklärung unverzichtbar.

Despite a reported patent foramen ovale (PFO) prevalence of 27.3% in the general population, perioperative paradoxical embolism in patients with PFO remains a rare but potentially catastrophic complication. Patients undergoing neurosurgical or joint procedures are at particular risk. During hip or shoulder arthroplasty, gaseous or particulate emboli can enter the venous circulation and result in clinically significant infarction. This risk can be further increased when bone cement implantation syndrome (BCIS) occurs concurrently. Using the presented case of a large right middle cerebral artery infarction as an example, we discuss the pathophysiological interplay between PFO and BCIS.

## Case study

The following case was submitted to a Medical Malpractice Review Board to assess whether medical malpractice had occurred.

An 81-year-old woman (height 163 cm, weight 54 kg) with therapy-resistant pain of the left hip due to grade IV coxarthrosis elected to undergo implantation of a partially cemented hip endoprosthesis. Her medical history included arterial hypertension, a patent foramen ovale (PFO) and two prior stroke episodes (1 and 5 years earlier) without residual neurological deficits. The PFO was classified as RoPE score 3 (Risk of Paradoxical Embolism [1–3]); therefore, prophylactic percutaneous closure was not performed. Preoperative transthoracic echocardiography demonstrated sinus rhythm, normal left ventricular systolic function (ejection fraction 65%), mild (grade I) mitral regurgitation, concentric left ventricular hypertrophy and moderate left atrial dilatation. No atrial thrombi were detected and there was no evidence of lower extremity deep vein thrombosis. Preoperative laboratory results were within normal limits. The patient was classified as American Society of Anesthesiologists (ASA) physical status III.

**Fig. 1:**
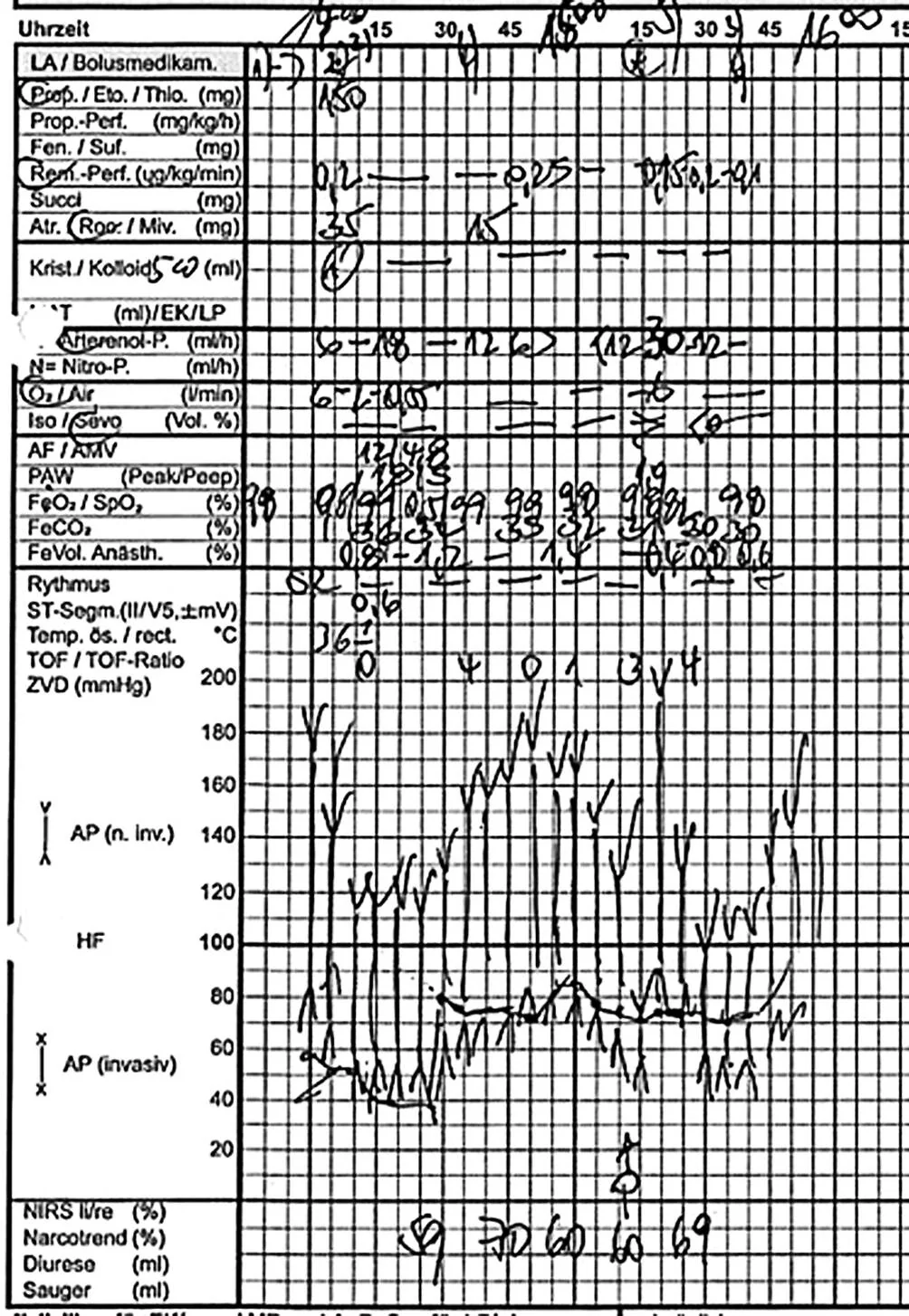
Excerpt of the handwritten anesthesia record

Following orthopedic and anesthesiological evaluation, an 18G peripheral intravenous cannula was placed on the dorsum of the right hand. Under standard monitoring (electrocardiography, ECG, noninvasive blood pressure, heart rate, SpO_2_, end-tidal CO_2_), anesthesia was induced and maintained with propofol, remifentanil, sevoflurane and rocuronium. Detailed information regarding the intraoperative management and the patient’s clinical course is provided in the anesthesia record shown in Fig. 1 and in the tabulated summary of the clinical progression presented in Table 1.

**Table 1:** Tabulated summary of clinical progression in 5‑min steps

Minutes	Action	BP (mm Hg)	HR (bpm)	SpO2 (%)	FeCO2 (mm Hg)	Drugs
*000*	Presence of anesthetists	–	–	99	–	2 g Cefazoline
*005*	Instrumentation	–	–	–	–	–
*010*	–	–	–	–	–	–
*015*	–	175/85	60	–	–	–
*020*	Start of anesthesia	145/70	50	98	–	150 mg propofol Start of 0.2 μg/kg/min remifentanil perfusion 35 mg rocuronium Start norepinephrine perfusion Start of crystalloid infusion Start of sevoflurane perfusion Illegible bolus
*025*	Preparing patient for surgery	115/60	50	–	–	4 mg ondansetron
*030*	–	115/60	40	99	36	–
*035*	–	115/60	40	–	–	–
*040*	–	115/60	40	–	–	0.5 mg atropine
*045*	Start of surgery	130/70	80	–	32	–
*050*	–	150/70	75	99	–	15 mg rocuronium
*055*	–	155/70	75	–	–	–
*060*	–	160/75	75	99	35	–
*065*	–	170/80	75	–	–	Stop norepinephrine perfusion
*070*	–	165/80	80	–	–	–
*075*	–	165/80	85	99	32	–
*080*	–	145/75	80	–	–	Start norepinephrine perfusion
*085*	PMMA (Palacos)	125/70	75	98	–	–
***090***	**–**	**75/44**	**70**	**82**	**31**	**2** **ml cafedrine/theodrenaline** **norepinephrine bolus**
*095*	–	190/90	75	98	–	–
*100*	–	130/85	75	–	30	–
*105*	–	100/60	70	–	–	–
*110*	–	100/60	70	–	–	4.5 mg piritramid
*115*	–	100/60	75	99	30	0.5 mg atropine
*120*	Suture	130/70	80	–	–	Stop remifentanil perfusion
*125*	End of surgery	145/80	90	–	–	–
*130*	–	–	–	–	–	–
*135*	End of anesthesia	–	–	–	–	–

*BP *Blood Pressure, *HR* Heart Rate, *SpO*_*2*_ Saturation of Peripheral O_2_, *FECO*_*2*_ Fraction of Exhaled CO_2_, *PMMA* Polymethylmetacrylate, *palacos*® German Brand Name for PMMA

As illustrated in Fig. 1 the readability of the handwritten anesthesia record was limited. Consequently, the precise chronological allocation of documented events and interventions, as outlined in Table 1, could only be reconstructed approximately.

The anesthesia record contains only one remark regarding the mode of ventilation: Pressure-controlled ventilation with positive end-expiratory pressure (PEEP, 18/5 cmH_2_O), respiratory rate 12 breaths/min, minute ventilation 4.8 l/min. The volume of intraoperatively administered crystalloid is not documented; however, it was certainly well below 500 ml. After extubation the patient was transferred to the post-anesthesia care unit (PACU).

In the PACU, the patient initially remained respiratory and hemodynamically stable while receiving the remaining crystalloid infusion. The following sequence of events, demonstrated in Table 2, was reconstructed from the medical records.

**Table 2:** Sequence of events in the PACU

Time (min)	Clinical course
000	Admission to the recovery room. SpO_2_ 93%, BP 155/80 mm Hg; 1 g paracetamol i.v.
020	Patient opened eyes to verbal stimuli
030	Patient remained drowsy upon verbal stimulation but demonstrated adequate spontaneous respiration and stable hemodynamics; nasopharyngeal tube was inserted
050	Hemoglobin concentration 11.2 g/dl (intraoperative blood loss was estimated as 300 ml); SpO_2_ decreased to 46%, supplemental oxygen was initiated via face mask at 3 l/min
075	Patient unable to respond adequately but opened eyes upon verbal stimulation; CCT was requested
121	CCT examination commenced
140	CCT findings were transmitted to the recovery room
150	Patient developed hypotension, BP 105/50 mm Hg; norepinephrine infusion was initiated
170	Propofol infusion was started; patient was reintubated
195	Invasive arterial blood pressure monitoring was established; BP 100/45 mm Hg
210	Following the diagnosis of an acute embolic infarction of the right middle cerebral artery with left-sided hemiparesis, the patient was transferred to a specialized clinic for further management

*SpO*_*2*_ Saturation of Peripheral O_2_, *BP* Blood Pressure, *CCT* Cerebral Computed Tomography

**Fig. 2:**
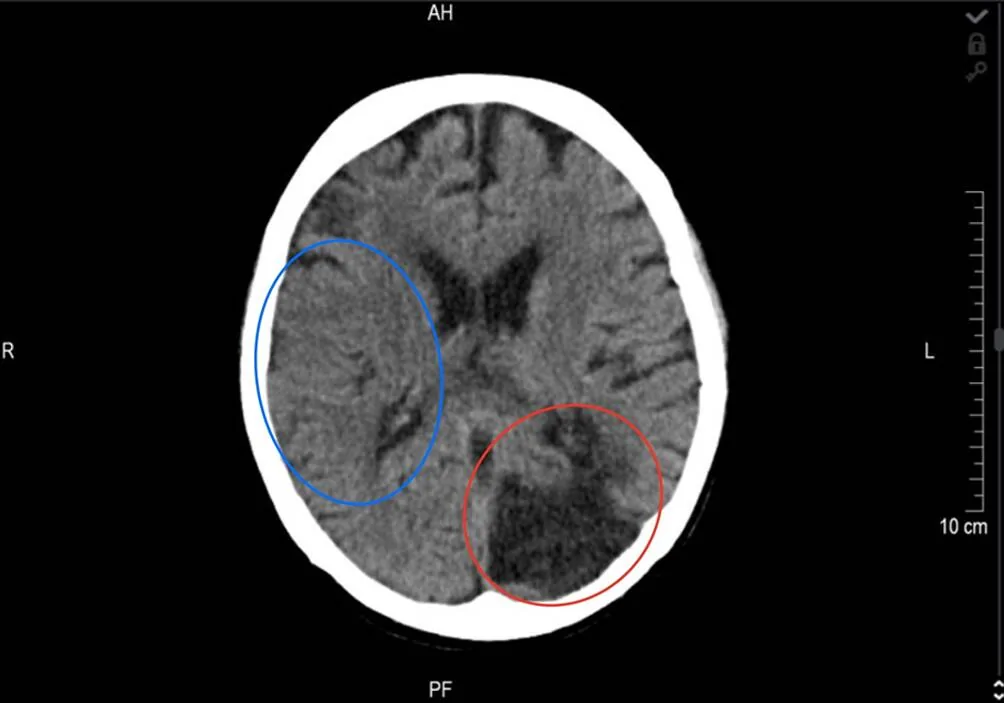
Cerebral Computed Tomography (CCT): Axial section at the level of the anterior horns of the lateral ventricles. For details, see text „CCT findings“

The radiological report described the CCT findings as (Fig. 2): subtle hypodense appearance of the white matter in the right frontoparietal region within the territory of the middle cerebral artery, with poor delineation of the head of the caudate nucleus and partially diminished corticomedullary differentiation as well as effacement of the cortical sulci compared to the contralateral side, consistent with early demarcation of an infarction in the right middle cerebral artery territory (blue circle). No evidence of hyperdensities suggestive of hemorrhage was visible. In the left parieto-occipital region, most likely chronic postischemic defects with adjacent gliosis are visible (red circle), resulting from the two prior stroke episodes 1 and 5 years earlier.

The patient’s daughter was contacted 4 years later and reported that the patient had been assigned care level 4 for home nursing care, indicating the most severe impairments in independence and functional abilities. The patient was able to ambulate independently using a walker; however, she suffered from pronounced visual disturbances, which the daughter was unable to describe in greater detail. At the age of 86 years, the patient had been admitted to a nursing home.

In the expert opinion, the medical expert assessed the treatment as negligent for the following reasons:

- Inadequate perioperative instrumentation, monitoring and therapy.
- Delayed postoperative evaluation of suspicious neurological symptoms.

To our knowledge, this is the first documented case describing the concurrence of paradoxical arterial embolism involving a large cerebral vessel, most likely secondary to BCIS.

## Pathophysiological aspects

Although it is plausible that BCIS could facilitate a paradoxical arterial embolism, this remains a pathophysiological hypothesis based on existing literature on BCIS and PFO. This plausibility can be inferred from published data on both entities [4–6]. The present case, to our knowledge, is the first to document the perioperative concurrence of a preoperatively known PFO, confirmed BCIS and a paradoxical cerebral embolism. In the following section, the potential pathophysiological links between these conditions are discussed in the context of the current literature on PFO and BCIS.

### Incidence of perioperative paradoxical embolisms

Whether a PFO increases the risk of cryptogenic stroke (embolic stroke of undetermined source, ESUS) remains controversial [2, 3, 6–8]; however, the risk of perioperative stroke appears to be higher in patients with a PFO than in those with an intact atrial septum, at least for certain surgical procedures [9, 10]. For example, Ng et al. reported more than a twofold increase in the risk of perioperative stroke among patients with confirmed PFO across various surgical indications [11]. Smilowitz et al. likewise described an approximately sixfold increase across multiple procedures [12]. In an analysis of more than 19,000,000 procedures, Villablanca et al. reported a stroke incidence of 5.9% in patients with an atrial septal defect (ASD)/PFO compared with 0.02% in patients with an intact atrial septum [13]. Rais et al. performed a meta-analysis of 11 studies including 21,250,546 surgical patients and found perioperative stroke rates of 14.59% in PFO carriers versus 0.38% in non-carriers [10].

Findings are less consistent in cohorts undergoing joint arthroplasty. Gai et al. reported postoperative neurological abnormalities in 1 of 202 patients undergoing knee or hip arthroplasty, of whom 16 (8%) were identified as PFO carriers [14]. Bido et al. observed no perioperative embolic events in a comparable cohort of 160 patients with ASD/PFO [15]. By contrast, Perfetti et al. reported a 29-fold increase in risk among 252 ASD/PFO carriers undergoing hip surgery compared with controls with an intact atrial septum [16]. These apparently divergent results may reflect the fact that not all paradoxical arterial emboli become clinically apparent. Clinical manifestation likely requires (i) an embolic burden exceeding a qualitative and quantitative threshold and (ii) embolization to a vascular territory associated with overt neurological deficits [17]. Consistent with this, Edmonds et al. detected intraoperative embolic signals in the central nervous system in 8 of 20 patients undergoing total hip arthroplasty, yet none developed clinically evident postoperative neurological symptoms [18].

### PFO

Because a PFO is typically asymptomatic throughout life despite a prevalence in the double-digit percentage range, it is most often detected incidentally during evaluation of cryptogenic stroke or at autopsy [17]. In an autopsy series of individuals of similar age to the patient described here, a PFO with a mean diameter of approximately 5 mm was reported in 20.2% of cases [19].

A PFO results from incomplete fusion of the septum primum and septum secundum. Under physiological conditions, the normal interatrial pressure gradient of approximately 2–4 mm Hg (left atrial pressure, LAP, greater than right atrial pressure, RAP) typically produces a small left-to-right (L/R) shunt, if any. Under certain circumstances, however, this gradient can be reversed, resulting in a right-to-left (R/L) shunt and thereby enabling paradoxical arterial embolism. Such a shunt reversal is usually triggered by a transient or sustained increase in pulmonary vascular resistance (PVR).

An increase in PVR elevates right ventricular afterload. If right ventricular function becomes impaired, RAP rises due to venous congestion, while reduced pulmonary blood flow decreases left atrial filling and can lower LAP. In the presence of a PFO, the resulting reversal of the atrial pressure gradient promotes R/L shunting and increases the risk of paradoxical embolization.

Several intraoperative factors can increase PVR. Even controlled ventilation with positive end-expiratory pressure (PEEP) can reduce pulmonary blood flow and thereby raise PVR [15, 17, 20, 21]. Pulmonary embolism due to embolization from lower extremity deep vein thrombosis (DVT) can also lead to clinically significant increases in PVR [6, 20]. In addition, during intracranial procedures and joint arthroplasty, gaseous or particulate emboli of varying size may enter the pulmonary circulation from the surgical field. Air embolism is a predominant mechanism in neurosurgery, particularly in the semi-sitting or sitting position [22]. In endoprosthetic surgery, in contrast, particulate emboli (e.g., bone, fat, wound debris and cement) are thought to play a major role [4, 5, 23–25]. Regardless of the underlying cause, any increase in PVR can precipitate R/L shunting in patients with a PFO.

### BCIS

Particulate emboli released during total hip arthroplasty, particularly during femoral preparation, prosthesis insertion and cementation, can precipitate severe hemodynamic and respiratory disturbances collectively termed bone cement implantation syndrome (BCIS). Reported incidences vary widely in the literature, ranging from 0% to 68% [5, 14, 15, 23–28]. Donaldson et al. described BCIS as a clinical syndrome characterized primarily by increased pulmonary vascular resistance (PVR), hypoxemia, hypotension, and cardiac arrhythmias (severity grades 1 and 2), which can progress to cardiovascular collapse (severity grade 3) [29].

The pathogenesis of BCIS remains incompletely understood and is thought to be multifactorial. In addition to embolic phenomena originating from the surgical field, proposed mechanisms include reflex pulmonary vasoconstriction due to cement-related release of vasoactive mediators and complement activation [5, 25, 28–30]. On this basis, cementless fixation has increasingly been advocated, as it may reduce cement-related mediator effects and potentially mitigate cerebral complications [23, 24, 31]; however, the risk of BCIS is not eliminated as gaseous and particulate emboli can still arise from the operative site; consequently, some authors have suggested that the term BCIS may be a misnomer [4, 32].

From a pathophysiological standpoint, it stands to reason that BCIS increases the likelihood of paradoxical arterial embolism. In the setting of a PFO, an acute rise in PVR due to pulmonary embolization from the surgical field can increase right-sided cardiac pressures and promote reversal of the interatrial shunt from left-to-right to right-to-left, thereby enabling systemic embolization. The potential clinical relevance of this mechanism is supported by a meta-analysis by Lucas et al. [6]. Among 1197 patients with pulmonary embolism, 318 (26.5%) had a PFO; stroke occurred in 19.5% of patients with PFO compared with 4.5% of those with an intact atrial septum.

Clinically, BCIS is diagnosed primarily on the basis of characteristic intraoperative changes, depending on severity, including hypoxemia (SpO_2_ < 94%), hypotension (greater than 20% decrease in systolic blood pressure), a decrease in end-tidal CO_2_ and tachycardia [4, 5, 23–25, 29, 30, 32]. In awake patients, impaired consciousness can also occur [29, 30, 33–35]. Although the precise mechanisms of BCIS remain to be fully elucidated, embolic obstruction appears to be a key contributor [4, 27]. Clinically, BCIS most closely resembles acute pulmonary embolism with respect to hemodynamic and respiratory manifestations [5]. Partial obstruction of the pulmonary circulation disrupts ventilation-perfusion matching, leading to hypoxemia (via increased intrapulmonary right-to-left shunting) and hypocapnia (via increased physiological dead space). Reduced transpulmonary blood flow decreases cardiac output, resulting in hypotension that can be partially compensated by an increase in heart rate. The extent to which additional mechanisms, such as complement activation, modulate the clinical phenotype remains uncertain [4, 29, 30].

### Coincidence of PFO and BCIS

As noted above, the hypothesis that BCIS-associated shunt reversal increases the risk of paradoxical embolism appears pathophysiological plausible; however, this relationship has not been addressed in the literature to date, either in review articles [4, 5, 25, 29, 30] or in clinical studies [15, 23, 24]. In recent case reports describing cerebral complications after joint arthroplasty, a PFO was either confirmed [17, 34, 36–38] or suspected [35] but none reported intraoperative BCIS. In addition, transpulmonary cerebral embolization may occur even in the setting of an intact atrial septum [18, 39].

The present case provides, to our knowledge, the first clinical documentation consistent with this proposed pathophysiological interaction between PFO and BCIS. After exclusion of deep vein thrombosis and other possible arterial sources of embolism (e.g., atherosclerotic plaques in brain-supplying arteries or left atrial thrombus) based on the patient’s history and preoperative evaluation, the most plausible remaining explanation is that the large middle cerebral artery infarction resulted from paradoxical embolization originating from the surgical field, with BCIS promoting right-to-left shunting via the PFO. Bone cement embolism itself is unlikely, as cement emboli of a size sufficient to cause a large vessel territory infarction would be expected to be visible on computed tomography [40]. The composition of the embolus (fat, bone marrow, air, or wound debris) cannot be determined; however, this uncertainty does not alter the clinical consequence.

### Anesthesiological implications

Although both PFO and BCIS occur with double-digit prevalence, paradoxical embolism appears to be a rare complication of joint arthroplasty. Moreover, except for the present case, paradoxical embolic events reported in recent years have occurred without intraoperative BCIS [17, 34–38]. Given the potential severity of such events, the relevant question is which preoperative and intraoperative anesthesiological measures are reasonable to reduce the risk of this potentially life-threatening complication.

At present, there are no clear guidelines regarding the optimal anesthetic technique for joint arthroplasty [4, 15, 26–30, 41–46]. Regional anesthesia, general anesthesia and combined techniques appear broadly comparable, with only minor differences in reported outcomes. No studies have specifically compared anesthetic techniques with respect to paradoxical embolism. From a theoretical perspective, each approach has potential advantages and limitations. Changes in consciousness can be detected earlier under regional anesthesia in awake patients [33, 35, 37]. Respiratory factors may also be relevant: coughing or straining during spontaneous ventilation can transiently increase right atrial pressure (Valsalva-like maneuver) and promote right-to-left shunting, whereas positive-pressure ventilation with PEEP may increase pulmonary vascular resistance and similarly favor shunt reversal [15, 17, 20, 21]. At present, it is not possible to determine which anesthetic technique, if any, would be most suitable to reduce the risk of paradoxical embolism.

In patients without a known history of PFO, routine preoperative screening is not typically performed before joint arthroplasty, in contrast to recommendations for selected neurosurgical procedures [14, 22]. Given that this patient population is generally older and often multimorbid, comprehensive perioperative monitoring and vascular access are considered essential [25]. A minimum of two peripheral intravenous lines and invasive arterial blood pressure monitoring are commonly recommended, with central venous access considered on a case-by-case basis [5, 25, 26, 29, 47, 48]. Some authors advocate pulmonary artery catheterization [30]. The perioperative use of transthoracic (TTE) or transesophageal echocardiography (TEE) has been discussed but is not routinely recommended and is viewed cautiously in this context [14, 22, 30]. An overview of mandatory and optional perioperative instrumentation is listed in Table 3.

**Table 3:** Mandatory and optional perioperative instrumentation

*Mandatory*
Standard anesthesiological monitoring (e.g., ECG, BP, HR, SpO_2_, FECO_2_)
Two peripheral venous lines
Invasive arterial blood pressure
*Optional (case-by-case basis)*
Central venous access
Pulmonary venous catheter
Carotid Doppler ultrasound
Transthoracic/transesophageal echocardiography

*ECG* Electrocardiogram, *BP* Blood Pressure, *HR* Heart Rate, *SpO*_*2*_ Saturation of Peripheral O_2_, *FECO*_*2*_ Fraction of Exhaled CO_2_

If a PFO is known preoperatively, the question of elective closure before surgery may arise; however, closure carries its own risks, and neurological societies generally recommend it only when the estimated likelihood that a stroke is attributable to the PFO is high [1, 3, 7]. The RoPE score (range 1–10) was developed to estimate the probability that a cryptogenic stroke is PFO-related. Prophylactic closure is typically considered only with RoPE scores > 7. The patient presented here had a RoPE score of 3 and therefore did not meet the criteria for closure. Whether the RoPE score is suitable for estimating perioperative embolic risk has not been established.

Most publications addressing BCIS emphasize close coordination between surgeons and anesthetists [4, 5, 23–25, 27, 30, 47, 48]. Because this principle applies broadly to perioperative care and is not specific to BCIS, it is not discussed further here.

With respect to preventing or managing BCIS, the literature largely focuses on general supportive measures applicable to many intraoperative complications, including increasing the inspired oxygen fraction and stabilizing hemodynamics with intravenous fluids and, if required, vasoactive agents [4, 5, 25, 28–30]. In cases progressing to Donaldson grade 3 BCIS, resuscitation should be performed according to established guidelines [5].

Given the limited options for preventing BCIS and the possibility of paradoxical embolism even in the absence of BCIS [36], careful postoperative surveillance is essential. Patients should be monitored closely and continuously after surgery and any new neurological, respiratory, or hemodynamic abnormalities should prompt immediate evaluation with appropriate, differentiated diagnostic work-up [25, 26, 28, 36].

Our study has some limitations. The primary objective was to elucidate the apparent pathophysiological interactions between PFO and BCIS. Of course, this association cannot be derived with certainty from a single, poorly documented and treated case report. To confirm this connection, an intraoperative carotid Doppler ultrasonography and/or TTE/TEE would have been helpful.

## Practical conclusion

- Patients with PFO have an increased risk of perioperative cerebral arterial embolism because intraoperative conditions may promote reversal of the transforaminal shunt and enable right-to-left passage of embolic material.
- Patients undergoing joint arthroplasty and certain neurosurgical procedures may be at particular risk due to the potential for embolus formation at the surgical site.
- In joint surgery, BCIS may further increase the likelihood of paradoxical embolism; however, the underlying pathophysiology of BCIS has not been definitively established.
- Despite double-digit prevalence rates for both PFO and BCIS, the perioperative concurrence of these conditions with subsequent paradoxical embolism seems to be rare.
- Clinical manifestation requires an embolic burden exceeding a qualitative and quantitative threshold and embolization to a vascular territory associated with overt neurological deficits.
- Despite potentially severe neurological consequences, routine preoperative PFO screening is not standard practice, largely because perioperative stroke is uncommon.
- It has not yet been clarified whether the RoPE score is suitable for assessing perioperative stroke risk.
- The optimal anesthetic technique and evidence-based perioperative protective strategies remain undefined; however, comprehensive invasive monitoring should be considered essential.
- After joint surgery patients should be monitored closely, and new neurological, respiratory, or hemodynamic abnormalities should prompt immediate, targeted diagnostic evaluation.

## Data Availability

Data can be made available in anonymized form upon reasonable request.

## Declarations

For this article no studies with human participants or animals were performed by any of the authors. All studies mentioned were in accordance with the ethical standards indicated in each case.
